# Exploring the Microbiota of East African Indigenous Leafy Greens for Plant Growth, Health, and Resilience

**DOI:** 10.3389/fmicb.2020.585690

**Published:** 2020-11-19

**Authors:** Julian Taffner, Olivia Laggner, Adrian Wolfgang, Danny Coyne, Gabriele Berg

**Affiliations:** ^1^Institute of Environmental Biotechnology, Graz University of Technology, Graz, Austria; ^2^East Africa Hub, International Institute of Tropical Agriculture (IITA), Nairobi, Kenya; ^3^Nematology Section, Department of Biology, Ghent University, Ghent, Belgium

**Keywords:** plant microbiome, archaea, leafy green vegetables, PGPR, amplicon analysis

## Abstract

Indigenous leafy green vegetable crops provide a promising nutritious alternative for East African agriculture under a changing climate; they are better able to cope with biotic and abiotic stresses than cosmopolitan vegetable crops. To verify our hypothesis that the associated microbiome is involved, we studied archaeal and bacterial communities of four locally popular leafy green crops in Uganda (*Bidens pilosa*, *Solanum scabrum*, *Abelmoschus esculentus*, and *Gynandropsis gynandra*) and of four plant microhabitats (phyllosphere, root endosphere, rhizosphere, and soil) by complementary analyses of amplicon and isolate libraries. All plants shared an unusually large core microbiome, comprising 18 procaryotic families but primarily consisting of *Bacillus*, *Sphingobium*, *Comamonadaceae*, *Pseudomonas*, and one archaeon from the soil crenarchaeotic group. Microbiome composition did not differ significantly for plant species but differed for microhabitats. The diversity was, in general, higher for bacteria (27,697 ASVs/*H* = 6.91) than for archaea (2,995 ASVs/*H* = 4.91); both groups form a robust network of copiotrophic bacteria and oligotrophic archaea. Screening of selected isolates for stress and plant health protecting traits showed that strains of *Bacillus* and *Sphingomonas* spp. div. constituted a substantial portion (15–31%) of the prokaryotic plant-associated communities. Across plant species, microbiota were characterized by a high proportion of potential copiotrophic and plant-beneficial species, which was not specific by plant species. The use of identified plant-beneficial isolates could provide the basis for the development of consortia of isolates for both abiotic and biotic stress protection to improve plant and ecosystem health, ensuring food security in East Africa.

## Introduction

The adoption of sustainable agriculture practices is necessary in order to feed growing populations, especially in Sub-Saharan Africa, where food insecurity and malnutrition indices are the most alarming globally (Sikora et al., 2019). Sustainable agricultural practices are additionally important for biodiversity and ecosystem services, the maintenance of which is a global challenge and which is attracting increasing attention (Mariotte et al., 2018). Eastern African farming systems comprise a tapestry of crops and livestock, which is dominated by smallholders and characterized by low productivity (Vanlauwe et al., 2014). A key factor explaining this low productivity is the high pest and pathogen pressure, and consequent production losses due to bioconstraints (Venkateswarlu et al., 2012; Dean et al., 2012; CIAT; BFS/USAID, 2017). The application of synthetic, chemical pesticides is a common coping strategy by farmers, but often using inappropriate, adulterated, out of date products, or generic compounds against which resistance has long since built up (Coyne et al., 2019). Consequently, in these smallholder systems, excessive misuse of pesticides may prevail, especially on crops which are prone to diseases and pests, such as vegetables (James et al., 2010). The detrimental effects of pesticide misuse are mostly reported in relation to human and animal health, but there are important considerations for ecosystem health too (Rosenstock et al., 1991; al-Saleh, 1994; Lemaire et al., 2006). The challenge therefore, is how to reduce the use of, and reliance on, pesticides and simultaneously improve yields while maintaining, facilitating, or enhancing biodiversity in farming systems.

A range of options is available to reduce dependency on synthetic pesticides, while their suitability depends on prevailing circumstances (Bender et al., 2016; Sikora et al., 2019). With the domestication of crops, their commercial exploitation, and the focus on breeding for ever-higher yielding cultivars, there has been a concomitant loss of resistance to stress factors as well as a decrease in microbial diversity (Mariotte et al., 2018; Cordovez et al., 2019). In contrast, indigenous plants that are less commercially exploited and less highly bred, but locally produced or gathered from natural habitats even, tend to be more robust and resilient (Venkateswarlu et al., 2012). In addition to their nutritional value, such properties have led to an increased interest in indigenous leafy green vegetables in Africa, where there is a need to raise the daily nutritional intake (Cernansky, 2015). Preferred traits include high levels of protein, iron, and other valuable nutrients, as well as their ability to better withstand biotic and abiotic stresses, compared to popular non-native vegetables, e.g., kale and cabbage (Kumar et al., 2010; Bartolome et al., 2013; Onyango et al., 2013; Cernansky, 2015; Ronoh et al., 2019). Further, these plants have a short and adapted life cycle, resulting in a lower vulnerability to irregular rainfall due to climate change. Speculation remains, however, why these plants are so robust and whether this robustness can be transferred between crops.

Plant fitness is a phenotypic expression and genotypically determined but can be modified by external factors, such as their associated microbiota. Plants and their associated microbiota combined represent a functional unity, the plant holobiont (Vandenkoornhuyse et al., 2015). Plant species are host to a high diversity and complexity of microbial communities, which vary depending on various external influences (Yeoh et al., 2017). These microbial communities can influence plant growth, productivity, adaptation, and health (Bulgarelli et al., 2013; Berg et al., 2016). Modes of action include nutrient supply, plant hormone production, and antagonism toward pathogens (Berg, 2009; Lugtenberg and Kamilova, 2009). Plant-microbe interactions have largely focused on bacteria and fungi, although archaea are widespread and stable components of plant microbiomes (Hardoim et al., 2015; Moissl-Eichinger et al., 2018). They have the potential to directly interact with the host plant by supporting nutrient supply and growth promotion *via* auxin biosynthesis (Taffner et al., 2018, 2019), while antagonistic properties are not yet known (Moissl-Eichinger and Huber, 2011). Our hypothesis is that the microbiome strongly contributes to the fitness and health of the indigenous leafy green vegetables and that the archaeal community is an important component. Identification of key species within these communities may be crucial to develop suitable biologically based options toward increased robustness and health in crops, and consequently toward the sustainable improvement of smallholder crop production systems in rural areas of Africa.

This study was aimed at characterizing the microbial communities of four leafy green crops grown in rural, smallholder conditions in Uganda, including blackjack (*Bidens pilosa* L.), nightshade (*Solanum scabrum* Mill.), okra (*Abelmoschus esculentus* Moench), and spiderwisp [*Gynandropsis gynandra* (L.) Briq.] and to assess the role of the bacterial and archaeal community on plant health. To achieve this, we combined next-generation sequencing and characterization of bacterial isolates as well as screening for antagonism toward five phytopathogenic fungi, including species of the top 10 economically important crop pathogens worldwide (*Botrytis cinerea*, *Fusarium oxysporum*, *Fusarium verticillioides*, *Sclerotium rolfsii*, and *Verticillium dahliae*; Dean et al., 2012).

## Materials and Methods

### Experimental Design and Sampling Procedure

The leafy green vegetables blackjack, okra, nightshade, and spiderwisp were sampled in Kasangati, Uganda (0° 26' 33''N, 32° 36' 19''E) in April 2017. Four samples, each consisting of a single plant (blackjack, spiderwisp, and okra) or three individual plants (nightshade), were gently removed with the aid of a spade, placed in sealed air-tight plastic bags, stored in a cool box, and transferred to the laboratory; four bulked soil samples were also collected and stored in separate plastic bags. Soil parameters were analyzed by “AGROLAB Agrar und Umwelt GmbH” (Sarstedt, Germany). The soil texture was sandy loam with pH = 5.9, organic matter content of 3.7%, and nutrient contents of K = 413 mg kg^−1^, P = 86 mg kg^−1^, and Mg = 214 mg kg^−1^. In order to homogenize the samples, 3 g of the phyllosphere (plant leaves and stalks), 5–10 g root material with adhering soil, and 5 g of soil per replicate were physically mixed in a BagMixer (Interscience, St. Nom, France) with 15 ml of 0.85% NaCl. Samples of root-adhering soil are further called rhizosphere. To obtain root endosphere samples, root samples were further surface sterilized with a 4% sodium hypochloride solution (NaClO) for 3 min, washed four times with 0.85% NaCl, resuspended in 15 ml NaCl, and then physically crushed with a sterile mortar and pestle. Samples were centrifuged at 16,500 *g* for 20 min at 4°C, and DNA extracts were then stored at −70°C for further processing.

### Isolation and CFU Determination of Bacterial Strains

Bacterial strains were isolated according to the protocol of Bragina et al. (2012). Briefly, 100 μl of the 15 ml 0.85% NaCl suspensions of each microhabitat-sample were plated onto NBII agar (Sifin, Berlin, Germany) plates in dilutions ranging from 10^−2^ to 10^−5^, incubated for 5 days at 20°C, and number of colony forming units (CFUs) determined and equated to fresh weight of the samples. A total of 512 randomly selected CFUs were isolated and stored in 20% glycerol at −70°C for further characterization.

### Screening of Antagonistic Bacteria and Antifungal VOCs Production

The 512 bacterial isolates were each streaked onto a Waksman Agar (WA)-plate and exposed to a fungal pathogen, following the protocols of Berg et al. (2006). The fungal phytopathogens *B. cinerea*, *F. oxysporum*, *F. verticillioides*, *S. rolfsii*, and *V. dahliae* were obtained from the strain collection of the Institute of Environmental Biotechnology (Graz University of Technology, Austria). Screenings were performed in triplicate and evaluated according to their antagonistic activity against pathogens according to Wolfgang et al. (2019). Isolates with strong antagonistic activity were further tested for volatile organic compound (VOC) production, using a two-clamp VOC assay (Cernava et al., 2015).

### BOX-PCR Fingerprinting and Sequencing of Antagonistic Bacteria

BOX-PCR was performed to resolve bacterial genetic diversity, according to the protocol of Rademaker and de Bruijn (1997). Shortly, colonies of 20 bacterial isolates with strong antagonism against all tested pathogens were solubilized, transferred into glass-bead filled tubes, ribolyzed, and centrifuged. PCR amplification was conducted using the BOXA1R primer 5'-CTA CGG CAA GGC GAC GCT GAC G-3'. After separation by gel electrophoresis, resulting band pattern was compared with “Gel Compar II” V.5.1 (Applied Maths, Kortrijk, Belgium). Different isolates were further sequenced based on the 16S rRNA gene fragment and taxonomically identified by manual BLAST search.^1^

### Abiotic Stress Assays and Phosphate-Solubilization Tests

Bacterial isolates with antagonistic activity toward the tested phytopathogens were additionally screened for resistance to abiotic stress, including drought, salinity, and reactive oxygen, as well as their potential to solubilize phosphate, as described by Zachow et al. (2013). In reactive oxygen species stress assays, bacterial isolates were cultivated overnight in LB (Lennox) medium (Carl Roth, Karlsruhe, Germany). Overnight cultures (5 μl) were added to 96-well plates filled with 195 μl LB in 10 different concentrations of tellurite (1, 3, 5, 7, 9, 10, 13, 15, 18, and 20 μg/ml), and hydrogen peroxide (from 100 to 1,500 μmol in 200 μmol steps, 1,750–4,000 μmol in 250 μmol steps), respectively. Growth of each isolate was measured after 24 h incubation at 30°C under agitation in four replicates using a plate reader (Infinite 200, Tecan Trading AG, Switzerland) at a wavelength of 600 nm (OD_600_). For evaluation of the tolerated osmolarity level, bacterial isolates were cultivated in LB media with various NaCl concentrations (from 0 to 15% in steps of 1%). Growth was measured in four replicates after 24, 48, 72, and 144 h using the plate reader at 600 nm. In order to test for drought resistance, 20 μl of an overnight culture were dried under sterile conditions in a 96-well plate and were resuspended in 20 μl 0.9% NaCl after 1, 2, 5, 7, 14, 60, and 80 days. Further, 10 μl of the resuspended cells was dropped onto LB-agar plates in a dilution series, incubated and number of CFUs determined.

### Screening for Plant-Growth Promoting Activities

Growth-promoting activities of bacterial isolates were tested on tomato (*Solanum lycopersicum* L. cv. Moneymaker, Austrosaat AG, Austria) plants according to Zachow et al. (2013). Tomato seeds were primed with bacterial cultures derived from three NBII plates grown overnight at 30°C suspended in 20 ml sterile water and were incubated for 4 h under agitation. Number of CFU per ml and OD_600_ of the suspensions were determined before seed priming (Table 1). Two germination pouches per strain were prepared with 8–9 seeds each. After 15 days, plants were harvested, leaf and root fresh weight were recorded. Roots were further pestled, and suspensions were plated on NBII plates in a dilution series for CFU determination. Plant growth was analyzed using Mann–Whitney U test.

**Table 1:** Abiotic stress confrontation assays.

Strain-ID	Species	Origin	Drought	H_2_O_2_ (μmol/ml)	NaCl (24 h)	NaCl (48 h)	NaCl (72 h)	NaCl (6 days)	OD_600_ (ONC)	CFU/ml priming suspension	ΔRFW (%)	ΔSFW (%)	ΔGR
Soil-I-11	*Bacillus* sp.	Soil	Tolerant	0	0	0	8%	6%	1.8	10^13^	67	46	+
Soil-I-14	*Bacillus* sp.	Soil	Tolerant	2,000	5%	7%	7%	7%	1.6	4.2*10^14^	83	6	+
Soil-I-45	*Sphingomonas* sp.	Soil	Tolerant	0	0	0	8%	11%	2.3	3.2*10^14^	−11	11	+
ORE-30	*Sphingomonas* sp.	Okra root	Tolerant	100	0	0	8%	10%	2.6	>3*10^14^	0	−24	+
ORE-44	*Sphingomonas* sp.	Okra root	Tolerant	0	0	0	8%	11%	2.8	>3*10^14^	21	−10	0
NSRE-37	*Bacillus* sp.	Nightshade root	Tolerant	900	0	0	0	0	1.7	5*10^12^	88	56	+

Growth after desiccation was measured by CFU/ml: tolerant: CFU above 10^5^ after drought for 88 days. Reactive oxygen species test performed with hydrogen peroxide (H_2_O_2_): highest concentration of H_2_O_2_, the isolate could still tolerate. NaCl(x): maximum concentration (w/v) of NaCl in growth medium resulting in increased OD_600_ relative to the control after time *x*. Increase in root fresh weight (ΔRFW), shoot fresh weight (ΔSFW), and germination rate (ΔGR; +, increase; −, decrease; 0, no effect) relative to the corresponding control group.

### Isolation of Total-Community DNA and Illumina Sequencing

Community DNA pellets from each microhabitat of soil, rhizosphere, root-endosphere, and phyllosphere of the four leafy green crops were subjected to PCR-based barcoding. First, extraction of DNA pellets was conducted using “FastDNA Spin Kit for soil” (MP Biomedical, Eschwege, Germany). PCR-products were purified with GENECLEAN TurboTM Kit (MP Biomedicals, Eschwege, Germany), following the manufacturer’s instructions for genomic DNA. The bacterial PCR approach was carried out with the Illumina barcode universal bacterial primer set 515f-806r (Caporaso et al., 2011) and PNA Mix (Lundberg et al., 2013) to remove host plastid and mitochondrial DNA. In order to amplify the archaeal 16S rRNA gene, a nested PCR was performed using the archaea-specific primers 344f and 915r in the first PCR. In a second PCR approach, the modified primer pair S-D-Arch-0349-a-S-17/S-D-Arch-0519-a-A-16 (here 349f/519r; Klindworth et al., 2013) with an additional 10 bp primer-pad (TATGGTAATT/AGTCAGCCAG) and linker (GT/GG) was used, according to the protocols of the Earth Microbiome Project (Walters et al., 2016). In a third PCR, Golay barcodes were annealed (Hamady et al., 2008). All PCR reactions were conducted as previously described (Taffner et al., 2019). Bacterial and archaeal PCR reactions were conducted in triplicate, purified with the Wizard SV Gel and PCR clean-up system (Promega, Madison, WI, United States), and pooled to equimolarity. Sequencing was carried out by Eurofins MWG Operon (Eurofins, Ebersberg, Germany) with an Illumina HiSeq 2500 system.

### Bioinformatic Processing of 16S rRNA Gene Fragments

The generated 16S rRNA gene libraries were pre-processed using QIIME version 1.9.1 (Caporaso et al., 2010) and QIIME2 (version 2018.2, Bolyen et al., 2019). First, read quality was checked with FastQC,^2^ reads were joined, and barcodes were extracted in QIIME1. Sequences were demultiplexed using the q2_demux plugin and denoised using q2_dada2 (Callahan et al., 2016) Taxonomy was assigned using a naïve Bayes taxonomy classifier (Bokulich et al., 2018) implemented in QIIME2. For taxonomic assignment, SILVA reference data base version 128 was used for bacteria and Silva 16S Archaeal database (349af–519ar 99, otusversion 128) for archaea with a 97 and 99% similarity cut-off, respectively (Quast et al., 2013). Amplicon sequence variants (ASVs) assigned to mitochondria or chloroplasts were removed using taxonomy-based filtering. ASVs were aligned with q2_mafft (Katoh and Standley, 2013), and a phylogenetic tree was constructed with q2_fasttree2 (Price et al., 2010). For estimating diversity metrics, sequence tables were rarefied to 1,210 ASVs (archaea) and 7,444 ASVs (bacteria). For evaluating alpha diversity, Kruskal-Wallis test (all groups and pairwise), alpha rarefaction, Shannon and Faith’s phylogenetic diversity index (Faith, 1992) were calculated. Beta diversity was analyzed by principal coordinate analysis (PCoA) plots and ANOSIM based on phylogenetic distance metrics of weighted UniFrac distances (Lozupone et al., 2007) and visualized with the emperor plugin (Vázquez-Baeza et al., 2013). The ANOSIM test was performed with 999 permutations. To test for the influence of microhabitat and plant species, these variables were tested using the plugin Adonis (Anderson, 2001) for bacteria and archaea. To test for significant differences in abundances of identified antagonistic taxa, the bacterial dataset was analyzed using the LEfSe algorithm implemented in https://www.microbiomeanalyst.ca (Chong et al., 2020). The dataset was filtered using the default settings (minimum count for reads of 4, minimum prevalence in samples 20%, low variance filtered based on 10% interquantile range, LDA score = 2.0), rarefied to minimum library size and scaled using total sum scaling. Taxa were compared on family level between each plant species (all microhabitats combined) and soil, as well as within a single plant species between microhabitats. Cytoscape 3.3.0 software was used to visualize the bacterial distribution and network of the core genera (Shannon et al., 2003). ASVs, that were found in >75% of the plant samples, were assigned as interspecific core ASVs of the plant species. ASVs were assigned to genus level, and data of all four plants were combined. Taxa represented in ≥50% of samples across the dataset were assigned as intraspecific core genera. Abundant sequences with a low taxonomical resolution were additionally assigned by using the nucleotide BLAST search.^3^

### Nucleotide Sequence Accession Numbers

The 16S rRNA gene fragment amplicon library was submitted to the European Nucleotide Archive (ENA) and can be found under the accession number PRJEB39392.

## Results

### General Community Structure of Prokaryotes Associated With Leafy Green Vegetables

Sequencing of the 16S rRNA gene fragments originating from the phyllosphere, root-endosphere, rhizosphere, and soil of the leafy greens blackjack, nightshade, okra, and spiderwisp resulted in a total of 10,688,730 high quality bacterial reads and 2,692,299 archaeal reads. After taxonomy-based filtering of mitochondria and chloroplast sequences, the datasets comprised 9,795,981 bacterial reads and 2,663,458 archaeal reads, clustered in a total of 27,697 and 2,995 distinct ASVs, respectively. Unassigned sequences remained in the dataset because we expected a considerable and potentially important part of microbes to be still unknown to science.

**Figure 1:**
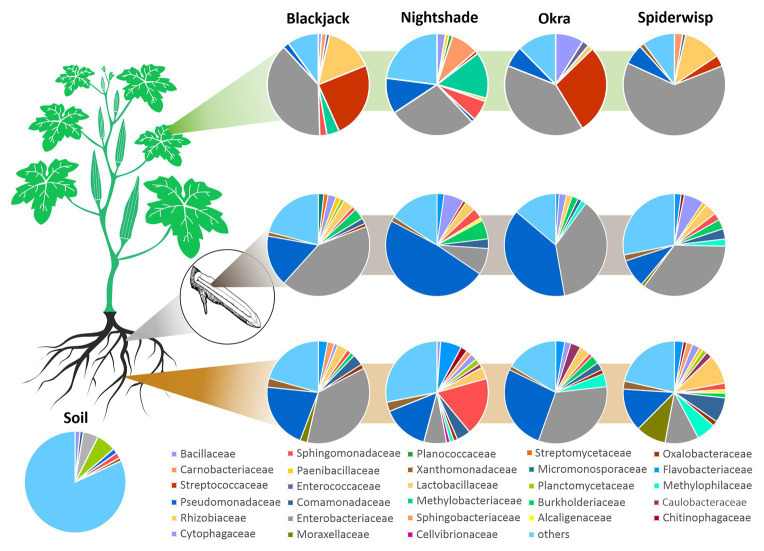
Bacterial core microbiome of leafy greens. The composition of the microbiome of blackjack, nightshade, okra and spiderwisp, and their microhabitats, displayed at the family level: phyllosphere (green stripe), root-endosphere (gray stripe), and rhizosphere (brown stripe). Families with abundances below 1% of total microbiome are captured within “others.”

The bacterial core microbiome revealed similarities and differences between the phytobiome composition in respect to the plant genotype and microhabitat (Figure 1). In the phyllosphere, *Enterobacteriaceae* (42.2%) and *Streptococcaceae* (14.4%) were dominant in the bacterial community, whereas in the root endosphere and rhizosphere *Enterobacteriaceae* (30.7 and 21.6%, respectively) and *Pseudomonadaceae* (28.0 and 19.0%, respectively) were predominant. In general, *Sphingomonadaceae* (4.2%), *Lactobacillaceae* (3.3%), *Bacillaceae* (2.9%), *Rhizobiaceae* (2.7%), *Comamonadaceae* (2.5%), *Flavobacteriaceae* (2.0%), and *Xanthomonadaceae* (1.5%) were ubiquitous but less abundant. In the phyllosphere of blackjack and Okra, *Streptococcaceae* were dominant, representing around a quarter of the core microbiome. Blackjack and spiderwisp both harbored *Lactobacillaceae* with 12.0–15.3% in the phyllosphere. *Bacillaceae* and *Pseudomonadaceae* were present in the core microbiome of each crop in each microhabitat (1.09–6.33%), with the exception in the spiderwisp phyllosphere, where no *Bacillaceae* were found. Throughout all microhabitats and crops, the fraction of families with an abundance lower than 1% (“others”) was relatively high (13.9–21.6%). These bacteria mainly belonged to the families *Oxalobacteraceae* (0.9%), *Caulobacteraceae* (0.9%), unidentified *Acidobacteria* (0.9%), *Sphingobacteriaceae* (0.8%), *Paenibacillaceae* (0.8%), *Rhizobiales* (0.7%), *Chitinophagaceae* (0.7%), *Planctomycetaceae* (0.6%), *Enterococcaceae* (0.6%), and *Alcaligenaceae* (0.5%).

**Figure 2:**
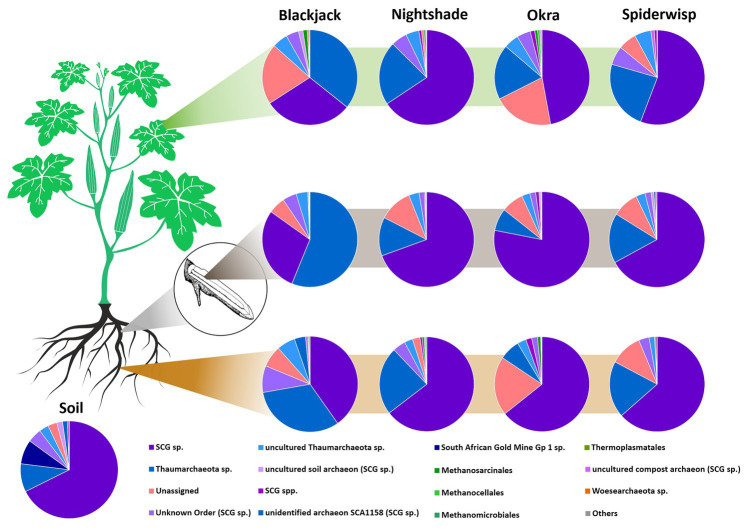
Archaeal community in leafy greens. The composition of the archaeal community of blackjack, nightshade, okra and spiderwisp, and their microhabitats, displayed at the order level: phyllosphere (green stripe), root-endosphere (gray stripe), and rhizosphere (brown stripe).

The archaeal communities (Figure 2) were clearly dominated by the phylum *Thaumarchaeota* (89.0%). In general, a high proportion of unassigned reads of up to 20.7% was detected, which were especially associated with blackjack and okra phyllospheres. In all four leafy green crops, *Euryarchaeota* were present but in low relative abundances (0.7–1.0%), except in spiderwisp, in which no *Euryarchaeota* were detected. At the class level, archaea of the soil crenarchaeotic group (SCG) were relatively abundant (56.2%), followed by unassigned *Thaumarchaeota* (22.9%). Archaea of the SCG were especially abundant in nightshade and spiderwisp. Methanogenic archaea of the class *Methanomicrobia* were mainly found in phyllosphere and the root-endosphere samples, except in spiderwisp.

### Bacterial Diversity Associated With Leafy Green Vegetables

Diversity metrics based on phylogeny were calculated with QIIME2 to determine similarities and dissimilarities of the bacterial community of the leafy green crops. Shannon’s diversity in the plant-microhabitats decreased from rhizosphere to phyllosphere, with an exception for nightshade, which showed the least diversity in root endosphere. However, diversity in the rhizosphere of nightshade was the highest comparing all plants [*H*_(nightshade)_ = 7.81 ± 0.21]. Diversity within the bulk soil samples was higher [*H*_(soil)_ = 9.41 ± 0.42], however, compared to the plant species [*H*_(mean)_ = 6.91 ± 0.16; ranging from *H* = 5.31 to *H* = 6.24].

**Figure 3:**
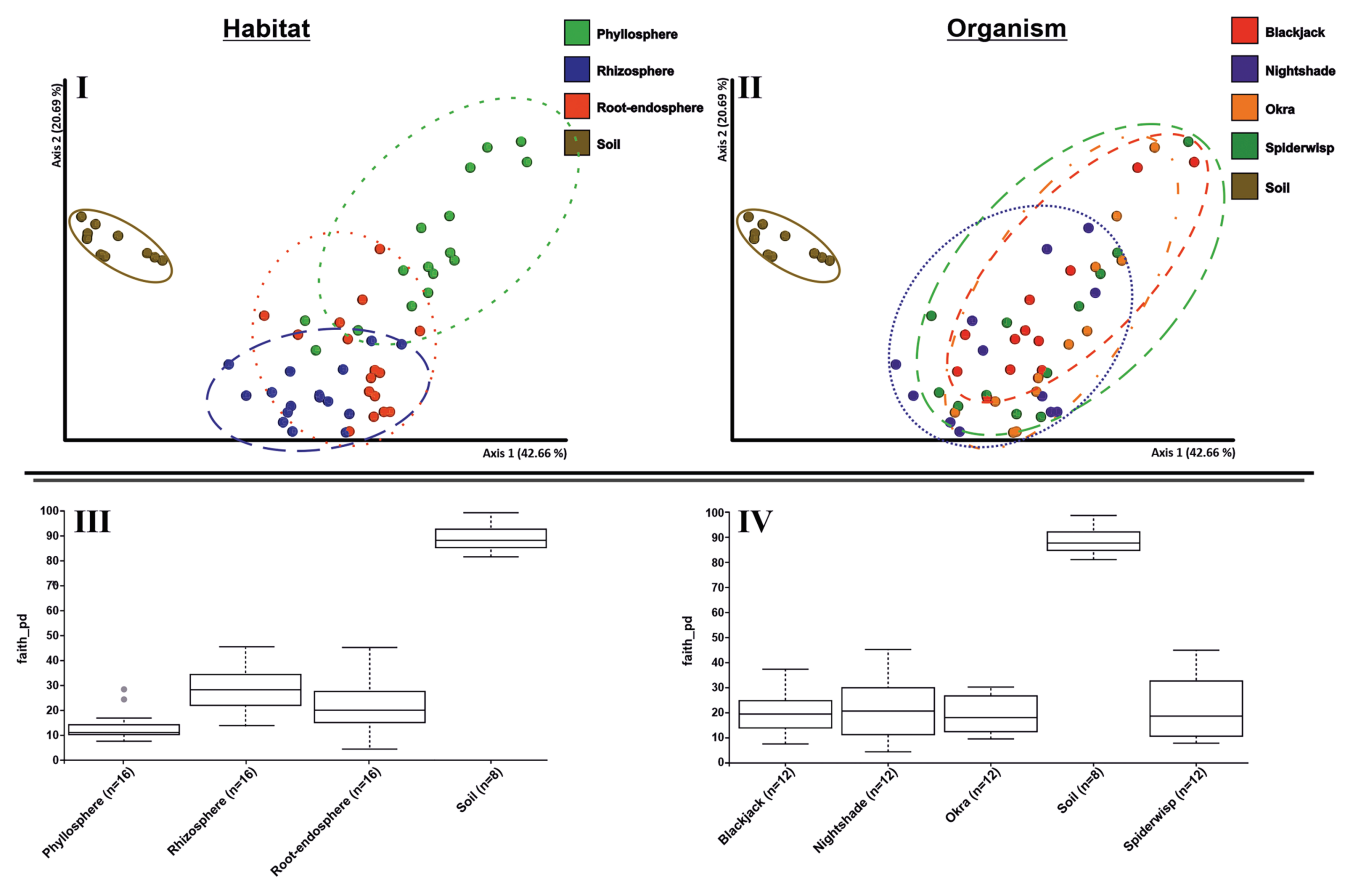
Bacterial alpha and beta diversity of leafy green vegetables. Principal coordinate analysis (PCoA) plots of the 16S rRNA amplicon datasets of four crops (blackjack, nightshade, okra, and spiderwisp) constructed based on phylogenetic distance metrics (weighted UniFrac). The distance between the data points negatively correlates with the similarity of the communities. **(I)** Clusters of the communities based on microhabitat (phyllosphere, rhizosphere, root-endosphere, and soil), and **(II)** based on plant species (blackjack, nightshade, okra, spiderwisp, and soil). Comparison of bacterial alpha diversity based on Faith’s phylogenetic diversity of the microhabitats **(III)** and plant species **(IV)**.

Between microhabitats, a cluster formation (Figure 3I) as well as a trend from rhizosphere to phyllosphere, was observed, whereas the rhizosphere bacterial community overlapped, to some extent, with the root endosphere. However, phyllosphere communities were more distinct, while the soil showed a clear cluster, which was significantly different to the other microhabitats with quantitative measures (ANOSIM: *R* = 0.504 and *p* ≤ 0.001). When assigning the same communities to their respective plant species (Figure 3II), no distinct clustering could be detected (ANOSIM: *R* = 0.048 and *p* = 0.064). Only nightshade had a slightly different clustering pattern. When investigating relationships within and between plants (within-sample), the alpha diversity index was significantly different between microhabitats (Figure 3III; *p* = 0.001), but not between the four plant species (Figure 3IV; *p* = 0.080). The overall group of microhabitats differed in diversity, but with respect to pairwise investigations, this was due to differences in rhizospheres as well as root-endosphere to phyllosphere. Further, group statistics showed that bacterial alpha diversity of the tested leafy green crops was not plant species specific. However, PCoA and Kruskal-Wallis test revealed that microbial diversity was microhabitat-specific. The factor “habitat” explained more variance within the bacterial dataset than the factor “organism” in both Bray-Curtis and weighted UniFrac distances (Supplementary Table 1).

### Archaeal Diversity Associated to Leafy Green Vegetables

**Figure 4:**
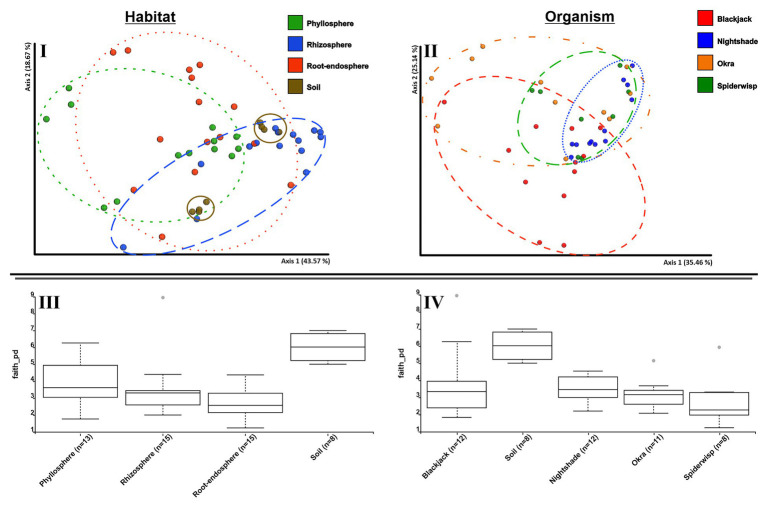
Alpha and beta diversity analyses of archaeal communities associated to leafy green vegetables. PCoA plots based on weighted UniFrac distance metrics show the archaeal community of phyllosphere, rhizosphere, root-endosphere, and soil microhabitats **(I)** of the four leafy green crops blackjack, nightshade, okra, and spiderwisp **(II)**; comparison of alpha diversity based on Faith’s phylogenetic diversity of the microhabitats **(III)** and plant species **(IV)**.

Archaeal alpha diversity indices had similar values in all plant species [*H*_(all)_ = 4.51–4.95], with the highest archaeal diversity in nightshade [*H*_(nightshade)_ = 4.95 ± 0.21]. Within plant-associated communities, the diversity of the microhabitats differed only slightly, between the root-endosphere [*H*_(endosphere)_ = 4.42 ± 0.37] and the rhizosphere [*H*_(rhizosphere)_ = 4.92 ± 0.23; Figure 4III]. Alpha diversity of archaeal communities in bulk soil was higher than in plant-associated communities [*H*_(soil)_ = 5.26 ± 0.27; Figure 4IV].

The alpha and beta diversity indices of the archaeal community are presented in Figure 4. In a PCoA-plot (Figure 4I), soil and the rhizosphere communities were clustered, whereas diversities in the root endosphere and phyllosphere were more dispersed. Again, a pattern, from rhizosphere to phyllosphere, was evident, as indicated by the overlapping clusters, with the soil diversity occurring within the rhizosphere diversity. In general, microhabitats showed significant differences in diversity (ANOSIM: *R* = 0.226; *p* = 0.001), with soil showing the highest diversity (Figure 4III). Analyzing the beta diversity for plant-type-specific differences, a cluster formation of nightshade and spiderwisp could be seen (Figure 4II). These plant species specific differences were confirmed by ANOSIM-test (*R* = 0.131; *p* = 0.002) and were found to be due to nightshade and blackjack (*q* < 0.05) based on pairwise comparison. However, spiderwisp and okra showed similarities (*q* > 0.377). Further, alpha diversity analysis with Kruskal-Wallis (all groups and pairwise) confirmed that archaeal diversity differed, depending on the microhabitat (*p* = 0.001) as well as the plant species (*p* = 0.01), which is due to the significantly different diversity of nightshade (Figures 4III,IV). However, pairwise comparison did not establish any differences between plant species (*q* > 0.08). Soil archaeal diversity was significantly different to all plant-associated microhabitats (*q* < 0.004), as well as the phyllosphere to root-endosphere (*q* = 0.038). However, the factors microhabitat and plant species explain <35% of the variance within the archaeal dataset (Supplementary Table 2), indicating other important factors determining archaeal community composition.

### Analysis of the Core Microbiota of Leafy Green Vegetables

**Figure 5:**
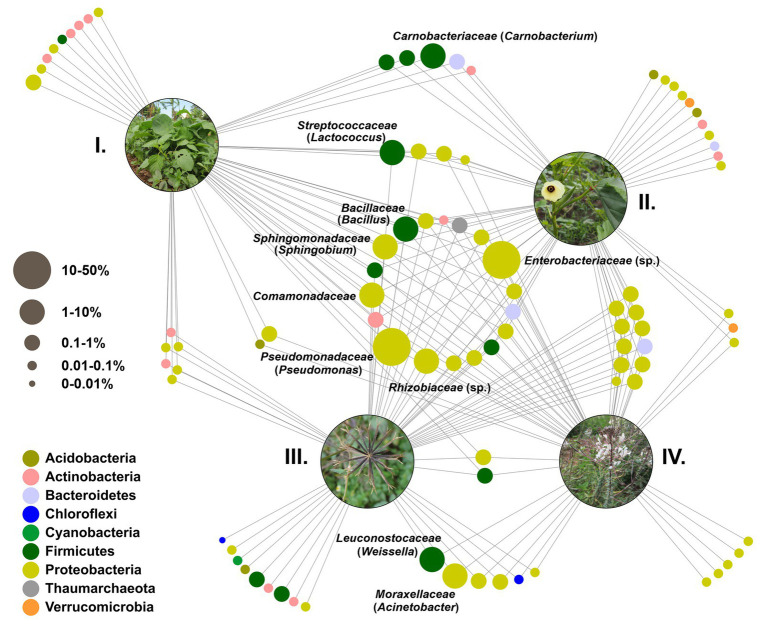
Feature network based on taxonomic analysis at the genus level. Each node represents a family of the core microbiome and is colored according to its phylum. If families were only represented by one genus within the core microbiome, the corresponding genus is added in brackets. Cross-linked nodes express families shared between the plants blackjack, nightshade, okra, and spiderwisp. **(I)** Nightshade. **(II)** Okra. **(III)** Blackjack. **(IV)** Spiderwisp.

Microbial core communities across blackjack, nightshade, okra, and spiderwisp were cross-linked based on taxonomic analysis at the family level and were visualized as a network (Figure 5). In total, 91 features were identified on genus level, with just one belonging to *Archaea*. A large core microbiome of 18 families, such as *Bacillus*, *Sphingobium*, *Comamonadaceae* gen., *Pseudomonas*, and *Rhizobiaceae* gen. (including the archaeal SCG), mainly assigned to *Proteobacteria*, were shared between all four crops. An additional 11 families, also mostly *Proteobacteria*, were common in blackjack, okra, and spiderwisp, thus communities associated with nightshade were more specific. Nightshade and okra shared specific taxa of the genus *Carnobacterium*, while blackjack and spiderwisp both shared *Weissella* and *Acinetobacter*. Each crop was associated with specific bacterial families that were unique in the core microbiome of the respective plant species. The number of such distinctive communities were ranged from five (spiderwisp) to nine (blackjack and nightshade) and 11 (okra).

### Screening and Identification of Bacterial Antagonists Against Biotic and Abiotic Stresses

**Figure 6:**
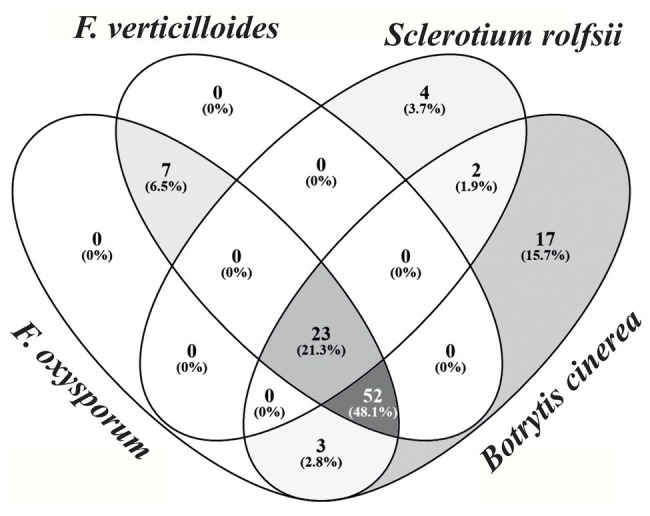
Number of bacterial isolates with antagonistic effects against fungal pathogens. Fungal pathogens included *Fusarium oxysporum*, *Fusarium verticillioides*, *Sclerotium rolfsii*, and *Botrytis cinerea*. Only bacterial antagonists showing high antagonistic activity were assigned to their respective fungi. Graph was generated using VENNY 2.1.0 (https://bioinfogp.cnb.csic.es/tools/venny/).

Of the 512 randomly selected bacterial isolates taken from the four leafy green crops and bulk soil, 108 isolates showed a high antagonistic activity (clear halo between fungi and bacteria ≥ 5 mm) against at least one pathogen (*B. cinerea*, *F. oxysporum*, *F. verticillioides*, *S. rolfsii*, and *V. dahliae*), and 23 isolates against four pathogens (Figure 6). Screening test results against *V. dahliae* needed a separate evaluation category as the culturing of the fungi required a different procedure and was, therefore, not included into the Venn diagram. A total of 44 bacterial isolates were highly active against *V. dahliae*. Based on these results, a selection of 24 antagonists, mostly antagonistic against all tested pathogens, were chosen for further characterization; 12 of the isolates originated from soil, nine were isolated from root endosphere, and three from the rhizosphere. Genetic characterization of the 24 antagonistic isolates undertaken using BOX-PCR and 16S sequencing identified 16 isolates as *Bacillus* sp. with suggested species *B. siamensis*, *B. velenzensis*, *B. amyloliquefaciens*, *B. methylotrophicus*, *B. vallismortis*, and *B. subtilis*. A further eight isolates were assigned to *Sphingomonas* sp. with hits for *S. echinoides* and *S. glacialis*. Combining the alignment results with similarity pattern of BOX PCR bands, isolates were clustered into five similarity groups (Supplementary Table 3).

The resistance to abiotic stresses of antagonistic bacterial strains was further characterized in order to evaluate their potential for application as future biocontrol agents (BCAs). Therefore, abiotic stress tests comprising reactive oxygen species stress tests were conducted (Table 1).

The desiccation assay showed that all tested bacterial isolates were highly resistant to drought with a CFU/ml of above 10^5^ after 88 days. The ability to resist reactive oxygen could not be shown in the tellurite assay, but when using hydrogen peroxide as stressor three isolates could still be cultured. Isolate “Soil-I-14” showed H_2_O_2_-tolerance as well as salt tolerance to high levels of NaCl after 24 h. Other isolates needed a longer period to adapt to higher NaCl concentrations and showed tolerance only after an adaption phase of 72 h (Table 1).

Further characterization of the mechanism of antagonism using two clamp VOC assays (TCVAs) showed no antagonistic effects of the bacterial isolates against the pathogens, based on VOCs.

### Plant Growth Promotion of Bacterial Antagonists

Priming of tomato seeds with the six bacterial antagonistic strains resulted in a significantly increased fresh biomass of leaves (*p* = 0.039) and whole seedlings (*p* = 0.020) relative to the control when using *Bacillus* strains. Priming with *Sphingomonas* species showed no significant effect on both root and leaves growth (Table 1). The strongest plant growth-promoting effect was observed when using a *Bacillus* strain derived from roots of nightshade (strain NSRE-37). None of the bacterial isolates showed signs of phosphate solubilization.

### Localization of Antagonists Within the Microbial Network of Leafy Green Vegetable Crops

**Figure 7:**
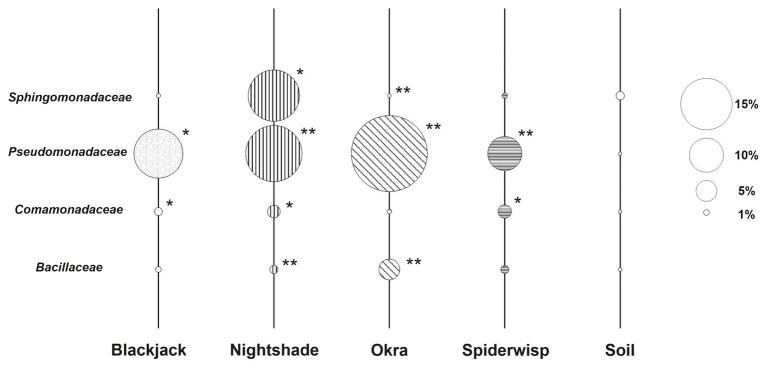
Relative abundance of antagonistic families of bacteria associated with leafy green vegetables. The diameter of the bubble represents the abundance of each family within the microbiome of each leafy green crop and soil. Soil is used as a reference. Families, which were found to produce nematicidal VOCs in Ugandan tomatoes (Wolfgang et al., 2019) – namely *Comamonadaceae* and *Pseudomonadaceae* – are included. Asterisks indicate significant differences in relative abundance compared to soil based on LEfSe (^*^p ≤ 0.05; ^**^p ≤ 0.001).

The distribution and abundance of bacterial families comprising taxa with high antagonistic activity toward fungal phytopathogens (*Bacillaceae* and *Sphingomonadaceae*) within the microbiome of the leafy green crops were compared in order to highlight possible links to the robustness of the plant host. Bacterial families that were isolated from Ugandan tomato and were shown to comprise nematicidal effects to plant-pathogenic nematodes in earlier studies (Wolfgang et al., 2019) were included. Most families were significantly enriched in plants compared to soil (Supplementary Table 4), although plant-specific differences were observed (Figure 7); while *Pseudomonadaceae* was the most abundant antagonistic family across all plant species with the highest relative abundance in okra, *Sphingomonadaceae* were higher abundant in soil except for nightshade. *Sphingomonadaceae* account for 9.6% (range 0.6–21.9) relative abundance in nightshade. Additionally, abundances of *Sphingomonadaceae* significantly differ within nightshade, with a higher relative abundance in rhizosphere and phyllosphere than in root-endosphere (Supplementary Table 5). Bacterial communities of nightshade consisted of the highest share of antagonistic families (31.2%), followed by okra (25.5%), blackjack (16.3%), and spiderwisp (14.8%). Within soil, antagonistic families comprised only 4.5% of all recorded microorganisms.

## Discussion

Research on plant-associated microbiomes in tropical regions is still in its beginning. Many factors known to influence crop-associated bacterial communities, e.g., soil quality, plants life cycle, or agronomic practices (Philippot et al., 2013), were not addressed in this study. Nevertheless, this study is a first step for understanding microbial communities in crops that are usually understudied in tropical agricultural research, but have a high relevance to local people. When investigating four locally popular leafy green vegetables (blackjack, nightshade, okra, and spiderwisp) in Uganda, we identified a microbiome that has both common and specific components between plant species. The general taxonomic composition was comparable with many other plant and crop species as well as the abundance of microbes (Bulgarelli et al., 2013; Berg et al., 2016). The rhizosphere was confirmed as the microbial hot spot for plants (Berg et al., 2006; Berg and Smalla, 2009) as well as the rhizosphere effect, which describes the selective enrichment visible in the composition of the microbiota (Foster et al., 1983; Buée et al., 2009). Interesting specific components were also observed. For instance, the microbiota of indigenous leafy greens were characterized by: (I) an unusually large core microbiome with only minor differences between plant species; (II) a high diversity of bacteria and archaea forming a network of potentially copiotrophic bacteria and oligotrophic archaea; and (III) a high proportion (15–31%) of potential plant beneficial microbes. The latter were identified in our culture collection, and can be potentially employed as biologically-based options for protection against stresses.

### Leafy Green Vegetables Harbor Common Bacteria With Copiotrophic Lifestyle

The diversity and community structure of bacteria and archaea in four leafy greens was found to be microhabitat-specific, rather than plant genotype-specific. The extent of the impact of numerous variables (e.g., plant genotype, plant organ, habitat, developmental stage, and soil quality) is a persistent question in microbial ecology across studies. However, in studies focused on natural vegetation in particular, the plant genotype seem to be the most important factors to determine plant-associated bacterial communities, followed by soil traits (Berg and Smalla, 2009; Bulgarelli et al., 2013). Recent studies revealed a decrease in diversity of crop-associated microbial communities through breeding practices (Cardinale et al., 2015; Mendes et al., 2019). The less pronounced impact of the plant genotype can be explained by the life strategy of plants. Three (blackjack, nightshade, and spiderwisp) of the four leafy greens in the current study were naturally occurring, and in general are ubiquitous, mostly invasive, produce many seeds and are, therefore, categorized as *r*-strategists, which often have a copiotrophic lifestyle (Andrews and Harris, 1986). This life strategy might also affect the composition of their associated microorganisms, which may have even co-evolved with them (Cordovez et al., 2019). For example, invasive plants, such as cheatgrass (*Bromus tectorum* L.), knapweed (*Centaurea stoebe* L.), and leafy spurge (*Euphorbia esula* L.), enrich copiotrophic bacteria in their associated rhizosphere (Gibbons et al., 2017). In our study, the microbiome associated with the leafy greens was neither specific nor depended on the plant genotype. We found that the most abundant bacterial phyla followed the same copiotrophic life strategy as their host, such as *Proteobacteria*, *Firmicutes*, *Bacteriodetes*, and *Actinobacteria* (Ho et al., 2017). In contrast, the archaeal phyla are considered to be oligotrophic, especially *Thaumarchaeota* (Uksa et al., 2015; Youssef et al., 2015). This indicates high substrate specificity and supports the assumption of a niche-colonization by archaea, including their role as followers of bacteria. Interestingly, a rhizosphere effect was also observed for archaea, especially for nightshade. The enrichment may depend on quality and composition of the root exudates, as demonstrated for archaea in tomato plants (Simon et al., 2005; Taffner et al., 2020). Altogether, copiotrophic bacteria and oligotrophic archaea appear to form a potent trophic network on leafy greens, which would warrant further investigation.

### High Microbial Diversity of Leafy Green Vegetables Compared to Cultivated Crops

The four leafy greens studied all showed high and relatively similar values for Shannon’s indices, with *H*-values ranging from 5.31 and 4.51 (okra) to 6.24 and 4.95 (nightshade), for bacteria and archaea, respectively. When comparing the Shannon’s indices in bacterial phyllosphere communities [ranging from *H*_(okra)_ = 4.4 to *H*_(nightshade)_ = 5.74], the diversity of the leafy greens in this study is considerably higher than in some commercially cultivated leafy greens, such as spinach [*Spinacia oleracea* L.; *H*_(spinach)_ = 3.15, Lopez-Velasco et al., 2013]. This also applies for rhizosphere: maize (*Zea mays*, L.) rhizosphere displayed a distinctly lower alpha diversity [*H*_(maize)_ = 3.42; García-Salamanca et al., 2013] than the diversity of the rhizosphere of the leafy greens [*H*_(mean_rhizosphere)_ = 6.91]. Further, the archaeal diversity in rhizosphere of leafy greens tested was found to be higher [*H*_(mean_rhizosphere)_ = 4.51–4.95] than in other cultivated crops, such as rice [*Oryza sativa*, L., *H*_(rice)_ = 4.08–4.43], Barbados nut [*Jatropha curcas*, L., *H*_(barbados_nut)_ = 3.16] and tomato [*H*_(tomato)_ = 3.4; Lee et al., 2015; Dubey et al., 2016]. This large disparity in microbial diversity is attributed to the overbreeding of our main crops (Pérez-Jaramillo et al., 2016), whereas natural leafy greens have received much less attention and remain less intensively bred, having only recently attracted interest in agriculture. Further, agricultural practices affect microbial diversity. Comparing organic farming with conventional intensive farming, significant differences in the microbiome of maize, melon (*Citrullus lanatus* Thunb.), pepper (*Capsicum annuum* L.), and tomato, as well as the soil, were prominent (Hartmann et al., 2015; Xia et al., 2015). Given the large difference in diversity indices between the uncultivated leafy greens from Uganda with intensively cultivated field crops, we could assume that highly focused breeding programs, as well as intensive agricultural practices have led to a reduction and loss of diversity in the microbiome of these crops (Pérez-Jaramillo et al., 2016). Naturally occurring vegetables, such as leafy greens, have a high microbial diversity, which is directly correlated with healthier, more robust plants that are less vulnerable to pathogenic outbreaks (Berg et al., 2016).

### High Proportion of Plant-Beneficial Bacteria of the Microbiome in Leafy Green Vegetables

A broad range of the taxa recovered from the core microbiome of leafy greens are well-known plant growth promoters, such as members of *Enterobacteriaceae* and *Pseudomonadaceae*, which occur frequently on leafy greens (Hayat et al., 2010). They are also known to be antagonistic against phytopathogenic fungi, either through competition or production of antimicrobial metabolites (Haas and Défago, 2005). However, *Enterobacteriaceae* also include human enteric pathogens, some of which, through occupation of crops *via* roots enter human digestive systems and have been associated with stimulating immune responses or acting as a “natural vaccination” as opposed a pathogen (Brandl, 2006; Berg et al., 2015). Further, *Actinobacteria* and *Proteobacteria* species were broadly distributed throughout the microbiome of the four leafy greens, both of which have previously been associated with host plant protection against fungal infections (Mendes et al., 2011). Besides the dominant families mentioned, some of the less common members of the microbiome showed growth promoting properties. *Bacillaceae* species such as *Bacillus subtilis*, *B. amyloliquefaciens* and *B. cereus*, and *Oxalobacteraceae* species such as *Herbaspirillum seropedicae* are known for supporting plant growth (Hayat et al., 2010), as well as members of the families *Xanthomonadaceae*, *Paenibacillaceae*, *Sphingobacteriaceae*, *Chitinophagaceae*, and *Alcaligenaceae* (Berg, 2009; Yang et al., 2014). One interesting fact is the relatively high abundance of *Sphingomonadaceae* in nightshade rhizosphere and phyllosphere (Figures 1, 7), compared to the other leafy greens. A high relative abundance of *Sphingomonadaceae* was frequently measured in other *Solanum*-associated communities, namely in rhizosphere (12%), root endosphere (5%), fruits (12–24%), and flowers (2–12%) of tomato (Allard et al., 2016; dataset of Wolfgang et al., 2019), rhizosphere of potatoes (*Solanum tuberosum* L., Pfeiffer et al., 2017), and rhizosphere of eggplant (*Solanum melongena* L., Li et al., 2019). *Sphingomonadaceae* comprise members with remarkable biotechnological potential, for instance degraders of aromatic or metalorganic compounds (Asaf et al., 2020). The high relative abundance of *Sphingomonadaceae* may be attributable to the diverse secondary metabolites (e.g., alkaloids) found in *Solanaceae*. However, further studies have to confirm the connection between *Sphingomonadaceae* and *Solanum*.

The archaeal community was clearly dominated by *Thaumarchaeota*, which are common colonizers of leafy greens, such as arugula (Taffner et al., 2019). This phylum consists mostly of ammonia oxidizing archaea (AOA), which are important for nitrogen cycling (Francis et al., 2007), and therefore, for supporting nutrient supplies to the plant. Further, recent studies show that archaea have the potential to directly support plant growth *via* auxin biosynthesis, a plant growth hormone (Taffner et al., 2018). Besides *Thaumarchaeota*, methanogens of the phylum *Euryarchaeota* were observed. These are also common in plants, colonizing anoxic niches in the rhizosphere, such as on maize or arugula (Chelius and Triplett, 2001; Taffner et al., 2019). However, there was a high relative abundance of taxonomically unassigned archaeal features, although an up-to-date established pipeline was used for the bioinformatic analysis. This limitation is well-known for archaea, especially in novel, less studied habitats such as Uganda, and is mainly due to poorly defined reference databases. We can conclude therefore that the core microbiome of leafy greens contained several taxa with the potential to support plant growth and protection against pathogenic fungi, and thereby contribute to the robustness and health of plant hosts.

### Promising Key Species for Future Biocontrol Agents

In the core microbiome of the leafy greens, we identified *Bacillus* spp. and *Sphingomonas* spp. playing a pivotal role in suppressing the key pathogenic fungi *B. cinerea*, *F. oxysporum*, *F. verticillioides*, *S. rolfsii*, and *V. dahliae*. *Bacillus* spp. have previously been shown to produce antimicrobial compounds, such as mycosubtilin and lipopeptides produced by *B. subtilis* (Leclère et al., 2005), or the antagonistic compound bacillomycin by *B. amyloliquefaciens* (Mülner et al., 2020). *Sphingomonas* are mainly known for their ability to degrade refractory contaminants, but have also been reported to be antagonistic against bacteria and fungi (White et al., 1996; Innerebner et al., 2011). These highly effective antagonists further showed resistance to abiotic stresses and plant-growth promotion capabilities in the current study. One isolate of *Bacillus* was able to tolerate high levels of hydrogen peroxide, which is a major abiotic stress factor for plants. Further, all isolates could grow under saline conditions up to 10%. Salinity reduces water-uptake efficiency and photosynthesis rate in plants, but microorganisms capable of dealing with such osmotic stress may confer resistance in plants to salt stress (Mayak et al., 2004). Furthermore, episodic drying and re-wetting of soil causes fluctuations in the soil’s water potential and challenges microbes. We showed that all our selected isolates were highly resistant to desiccation. Effective consortia of biological control agents, therefore, should include bacteria that support plant growth in addition to antagonistic species. In our study, priming of tomato seeds with *Bacillus* isolates resulted in significant plant-growth promotion of up to 70%, whereas *Sphingomonas* isolates did not show any effect. However, *Sphingomonas* are known to promote plant growth by producing gibberellic acids (GAs) and indole acetic acid (IAA), improving crop productivity, which have also been reported for *Bacillus* spp. (Khan et al., 2014). Although *Bacillus* strains have previously been reported as solubilizers of inorganic phosphate (Hayat et al., 2010), we could not identify phosphate solubilizers among our isolates. This provides confirmation of the high specificity of plant beneficial traits at strain level (Berg, 2009). The antagonistic and plant-growth-promoting characteristics of the *Bacillus* and *Sphingomonas* isolates tested make them promising candidates for their application as biological control agents against fungal infections and for increasing robustness and plant health in Ugandan agriculture.

## Conclusion

In our study, we found a unique, diverse, and robust microbiome occurring on natural leafy green vegetables in Uganda. Blackjack, okra, nightshade, and spiderwisp harbored microbes with strong antagonistic activities against pathogenic fungi, as well as promoting plant growth and demonstrating properties to enable host plants to withstand abiotic stresses. Six isolates in particular, assigned to the families *Sphingomonadaceae* and *Bacillaceae*, proved to be promising key-candidates for future sustainable biocontrol agents, toward supporting crop production in smallholder production systems in Sub-Saharan Africa. The biocontrol approach provides a more environmentally sustainable opportunity to produce crops and reduce or even replace excessive pesticide use. Identification of microbial isolates that are indigenous and adapted to African smallholder production systems will enable the development of technologies to support smallholders and improve human and environmental health.

## Data Availability Statement

The datasets presented in this study can be found in online repositories. The names of the repository/repositories and accession number(s) can be found at: https://www.ebi.ac.uk/ena, PRJEB39392.

## Author Contributions

JT, DC, AW, and GB performed the sampling. JT and OL conducted experiments in the laboratory and drafted the manuscript. JT, OL, and AW performed bioinformatics analyses. All authors contributed to the article and approved the submitted version.

### Conflict of Interest

The authors declare that the research was conducted in the absence of any commercial or financial relationships that could be construed as a potential conflict of interest.

## Footnotes

^1^https://blast.ncbi.nlm.nih.gov/

^2^http://www.bioinformatics.babraham.ac.uk/projects/fastqc/

^3^https://blast.ncbi.nlm.nih.gov/Blast.cgi
